# One or two? A Process View of pregnancy

**DOI:** 10.1007/s11098-021-01716-y

**Published:** 2021-09-15

**Authors:** Anne Sophie Meincke

**Affiliations:** Department of Philosophy, University of Vienna, Neues Institutsgebäude, Room C0210, Universitätsstraße 7, 1010 Vienna, Austria

**Keywords:** Pregnancy, Substance ontology, Process ontology, Parthood, Organism, Hylomorphism

## Abstract

How many individuals are present where we see a pregnant individual? Within a substance ontological framework, there are exactly two possible answers to this question. The standard answer—two individuals—is typically championed by scholars endorsing the predominant Containment View of pregnancy, according to which the foetus resides in the gestating organism like in a container. The alternative answer—one individual—has recently found support in the Parthood View, according to which the foetus is a part of the gestating organism. Here I propose a third answer: a pregnant individual is neither two individuals nor one individual but something in between one and two. This is because organisms are better understood as processes than as substances. With a special focus on the Parthood View, I explain why a Process View of pregnancy, according to which a pregnant individual is a bifurcating hypercomplex process, surpasses the substance ontological approaches.

## Introduction

How do we count pregnant individuals? Speaking of a ‘pregnant individual’ seems to suggest that the answer is already clear: if a pregnant individual is an *individual*, then this is to say that it is *one* individual.^1^ Individuals may have parts; but these do not count extra. My left kidney, for instance, is a part of me; but its numerical identity does not add to my numerical identity, which instead remains ‘one’; nor do I become less than ‘one’ if I donate my left kidney to somebody. If you, accordingly, think that exactly one individual is present where we see a pregnant individual at a certain time, then you are likely to be committed to the so-called Parthood View of pregnancy, i.e., the view that the foetus, or ‘foster’ (as I shall hereafter say),^2^ is a part of the pregnant individual, or ‘gravida’.^3^

If you, however, think that two individuals are present where we see a pregnant individual at a certain time, then you are likely to be committed to the so-called Containment View.^4^ According to this view, the foster resides in the gestating organism like in a container, i.e., is *merely* contained in the gestating organism as opposed to being a part of it.^5^ On the standard reading of the Containment View, this implies that the foster is a second individual that just happens to be hidden underneath the pregnant individual’s visible surface.

Note that there is no *necessary* link between the Containment View and the view that there are two individuals where we see a pregnant individual. For instance, it is possible to claim that the foster is merely contained in the gravida but that still there is only one individual present where and when we see a pregnant individual because the foster does not qualify as an individual in its own right. Yet, the Containment View and (as we may call it) the Two Individuals View typically occur in conjunction and, moreover, dominate our thinking about the metaphysics of pregnancy. This, I explain in Sect. 2 of this paper, is no accident but symptomatic of the predominance of substance ontology in Western philosophical thought in general and of the lasting impact of Aristotelian hylomorphist substance ontology on Western thinking about reproduction, pregnancy and embryogenesis in particular. It is because of (broadly) Aristotelian substance ontological assumptions that pregnant individuals are commonly viewed as containers of another individual—a view which, however, as we shall see, proves untenable for empirical reasons.

Note also that neither is there a *necessary* link between the Parthood View and the One Individual View. Again, the intuitive interpretation of the former as implying the latter indicates the impact of powerful underlying substance ontological assumptions. However, as I shall show in Sect. 3 of this paper, substance ontology causes problems for the Parthood View that weaken the latter’s ambitions to supersede the Containment View. Not only do we as of yet lack a satisfactory explication of the notion of parthood employed by the Parthood View, but most importantly this view—as developed in Kingma’s pioneering work (Kingma, 2018, 2019, 2020b)—struggles to square the parthood claim with the plausible assumption of numerical identity between the foster and the neonate without thereby running into difficulties with counting. While the Containment View is logically consistent but unconvincing on empirical grounds (Kingma, 2018), the Parthood View takes into account empirical facts but gets caught up in contradictions.

This situation calls for a radical new beginning and, that is, as I shall demonstrate in Sect. 4, for a fundamental rethinking of the metaphysics that underlies our views of pregnancy. What if organisms^6^ are not substances, as metaphysicians have traditionally thought, but processes, as a growing number of philosophers of biology (e.g., Dupré, 2012, 2020; Meincke, 2019a; Nicholson & Dupré, 2018) have come to maintain? I shall propose a novel Process View of mammalian^7^ pregnancy, according to which a pregnant individual is a bifurcating hypercomplex process and, hence, neither two individuals nor one individual but something in between one and two. I show that the Process View, by acknowledging the processual nature of organisms in general and of mammalian pregnancy in particular, overcomes the difficulties of the Parthood View. It marks out the foster as a *sui generis* part of the pregnant organism and secures identity between the foster and the neonate. Unlike both the Containment View and the Parthood View, the Process View captures the temporal dynamics of mammalian pregnancy.

## The Containment View of pregnancy

According to the Containment View, a pregnant individual is like a container for another individual. This implies that being located inside the gestating organism is not essential to the gestated organism’s identity. Indeed this bold claim has been part and parcel of traditional Western reflection on reproduction, pregnancy and embryogenesis within a substance ontological framework.

Substances are ontologically independent things, discrete particulars with well-defined boundaries and capable of persisting through change. According to Aristotle’s hylomorphist substance theory, each such substance is composed of matter and form, with the form defining the substance’s immutable essence. Strikingly, hylomorphism is a gendered story presenting the form as the active ‘male’ component and matter as the passive ‘female’ component. In conception the male semen is thought to contribute the form and, that is, essence of the embryo while the female merely contributes the matter through nourishing. Aristotle famously compared the foster’s relationship to the gestating organism to that of a plant feeding on a soil (Aristotle, 1963, *GA*, e.g., 739b 34–740a 1), thus denying any substantial contribution being made by the gestating organism to the foster’s development (Tuana, 1988; cf. Connell, 2016).

Thanks to the discovery of female gametes, we now know that the paternal and the maternal organism each contribute half of the genetic makeup of the foster. However, the basic assumptions of the so-called flowerpot model of pregnancy (Whitbeck, 1973/74) are still in place in the Containment View which treats the womb as a nourishing container while insisting that the foster, qua substance, develops into a recognisable exemplar of its species largely independently of external circumstances, in virtue of its essential form, entelechy or ‘intrinsic potential’ (Gómez-Lobo, 2005; Howsepian, 2008; Oderberg, 1997; Reichlin, 1997). As a result, discussions in metaphysics focus almost exclusively on ontological features of the foster (in particular, in the context of questions concerning the moral status of the human embryo), ignoring its relationship with the gravida. That the Containment View, despite its popularity, is rarely defended explicitly and worked out in detail—with the important exception of Smith and Brogaard (2003)—lies in the very nature of this view. As a mere container, the gravida disappears from the horizon of investigation.

Kingma (2018, 2019, 2020b) has recently provided empirical arguments against the Containment View which in turn, she claims, support the Parthood View. A closer look at embryology reveals that the foster, rather than being a neatly bounded entity with its own independent identity,^8^ is in fact strongly intertwined with the gravida in at least four respects, all of which are being discussed in the philosophy of biology as criteria of biological individuality: (1) the foster belongs to the internal environment of the gravida which is actively maintained by the gravida in a state of homeostasis, and it relies on the gravida for many of its important physiological functions; (2) the foster is functionally and metabolically integrated in the gravida which makes various (anatomical and metabolic) adjustments to facilitate the foster which, in turn, relies on the gravida for its metabolic functions; (3) the foster is topologically connected with the gravida through the placenta and the umbilical cord; (4) the gravida’s immune system is set up to actively tolerate, rather than reject, the foster (Kingma, 2019: 622ff.).^9^

Kingma adds that for the foster to be merely contained in the gravida—like a tenant in a niche or a tub of yoghurt in the fridge, as Smith and Brogaard (2003: 69ff. and 74) claim—it would need to be able to freely move in and out (Kingma, 2020b: 374f.). But this is exactly not possible. Being physiologically, functionally, topologically and immunologically entangled with the gravida, the foster can neither leave the womb whenever it may want to do, nor can it re-enter after leaving it. Birth (vaginal or by caesarean section) is how fosters get disentangled from the gravida, and it is irreversible: “once a baby is out, it does not go back in” (Kingma, 2020b: 375).

Kingma’s negative case against the Containment View is compelling. Given the intimate and multifaceted intertwinement between gravida and foster it is impossible to maintain the traditional container image.^10^ The foster is crucially dependent for its development on interactions with the gravida, as has been argued already against the idea of an ‘intrinsic potential’ to personhood supposedly possessed by human fosters (Meincke, 2015, 2018a).^11^ Empirical findings on reproduction, pregnancy and embryogenesis reveal the Containment View to be obsolete. If so, one may, of course, wonder about the appropriateness of the overall ontological framework on which the Containment View traditionally has relied: how suitable is substance ontology—an ontology of neatly separated, discrete things—to understanding pregnancy? We will return to this question after assessing the positive claims made by the Parthood View.

## The Parthood View of pregnancy

### In what sense are fosters parts of gestating organisms?

According to the Parthood View, the foster, instead of being merely contained in the gravida, is a part of it. How exactly are we to understand the parthood claim? Kingma (2019: 4f.) explains that the latter “employs our common-sense understanding of part-whole relations, an understanding according to which kidneys are parts of dogs, table-legs parts of tables, and engines parts of cars”. This statement is unhelpfully vague, given the rather diverse examples.^12^ It therefore comes as no surprise that Kingma, when arguing for the Parthood View, focuses on the first example: the sense in which kidneys are parts of dogs (Kingma, 2019: e.g., 4, 12, 29). This, however, prompts a new question. Kidneys are parts of dogs in the sense of being an organ of a particular organism. So are fosters then organs of gestating organisms?

Organs are homeostatically, physiologically, functionally and metabolically integrated in the organism, they are topologically connected with the organism and tolerated by the organism’s immune system. Organs, that is, are clear cases of parts of organisms according to the criteria of biological individuality invoked by Kingma. However, organs are special parts insofar as they perform specific functions for the organism of which they are a part. Typically, this means that they make some necessary contribution to the organism’s survival. We cannot say this of the foster, though. It is hard, or impossible, to survive without your kidneys; but it is no problem at all to survive without a foster in your womb. At least in modern Western societies, women do not have a foster inside their bodies most of the time of their lives, and the same appears to be true for most other female mammals too.^13^

There is an obvious reaction to this objection: the point of having a foster inside our body is not survival but rather reproduction, i.e., fitness or continuation of the lineage. Thus Kingma (2019: 626) reminds us that survival is just a means towards the end of reproduction, and explains that accordingly organs that promote the latter instead of the former cannot be excluded from being parts of organisms (see also Kingma, 2018: 182). Agreed—but should we conclude from this that fosters are parts of gestating organisms in the sense of organs serving the function of reproduction? Surely not. A foster is rather what results from reproduction as facilitated by reproductive organs. In other words, there is a clear distinction between (female) reproductive organs and fosters. Briefly put, the former produce; the latter are produced. This difference is arguably reflected in different temporal profiles: in higher organisms such as mammals, female reproductive organs (uterus, Fallopian tubes, ovaries, vulva, labia, clitoris), like most other organs, are stable; once developed during embryogenesis, they exist throughout the lifetime of the organism.^14^ Pregnancies, however, come and go. At least,^15^ organs, unlike fosters, do not usually leave the organism to continue existing on their own.

If fosters are not organs, what sort of ‘lady parts’ (Kingma, 2018), then, are they? Certainly, they are not limbs or body fluids. Nor are they comparable to the appendix, a presumably^16^ merely vestigial and functionless body part. Admittedly, there is something functional about having a foster inside the womb for female mammals, without this implying that the foster is an organ of female mammals. The most accurate characterisation of the specific sense in which a foster is a part of a gestating organism thus seems to be this: a foster is a female body part that is functional to reproduction insofar as it is the result of reproduction (as opposed to facilitating it). This is in effect to say that a foster has the status of a *sui generis* part—something we may be happy to accept in the light of the *sui generis* character of reproduction. However, it leaves us with the task to spell out the specificities of this *sui generis* case of parthood.

If *sui generis*, the foster-meaning of parthood, rather than being analogous to the kidney-meaning of parthood, adds to the list of meanings of parthood specified by Kingma. Hence, we can exactly not rely on common-sense intuitions about the part-whole relation. Instead, fleshing out the notion of foetal, or ‘fostal’, parthood will require an account of what makes fostal mereology, or the mereology of mammalian pregnancy, special. Such an account seems perfectly possible. However, as things currently stand, the Parthood View of pregnancy deploys a notion of parthood that is either inappropriate, by likening fosters to reproductive organs, or too vague to help our understanding of the core claim of the Parthood View: that fosters are *parts* of gestating organisms.

### Are fosters (Substances that are) parts of substances?

Kingma (2019: 639) admits that the Parthood View needs further elaboration, noting that questions about how it is to be interpreted are related to more general ontological assumptions (see also Kingma, 2019: 612). In what follows I want to show that ontology indeed matters, by demonstrating how combining the Parthood View with the view that organisms are substances prevents a satisfactory interpretation of the Parthood View.

On the standard version of the Containment View that draws on Aristotelian substance ontology, there is no doubt that the foster is a substance in its own right. The foster, just like the gravida, instantiates a form or natural kind—for instance, the kinds ‘human being’ or ‘cat’. It does so from the moment of conception (which was Aristotle’s original claim, but see also, e.g., Oderberg, 1997, 2008; Reichlin, 1997; Damschen et al., 2006) or at least no later than about 14 to 16 days after conception (as claimed by some contemporary (broadly) neo-Aristotelian and neo-Thomistic scholars with respect to human fosters, e.g., Johnson, 1995; Smith & Brogaard, 2003).^17^ Mammalian organisms thus start existing at conception or about 14–16 days after conception. Accordingly, from this moment on there are two individuals where we see a pregnant individual.

When do mammalian organisms start to exist on the Parthood View? Kingma (2019: 630f.) argues that the four criteria of biological individuality, at least taken together, suggest that the foster ceases to be a part of the gravida at birth. While functional integration and metabolic unity tend to continue for some time after birth, mother and offspring are distinct individuals with respect to homeostasis and physiology, topology and (with some restrictions) immunology. If organisms are substances, and assuming—as substance ontologists traditionally have done—that parts of substances are not substances themselves, it follows that the foster is not a substance in its own right and, hence, that mammalian organisms come into existence at birth. Up until a pregnant individual gives birth to a live offspring, there is exactly one individual where we see a pregnant individual.

Exploring possible strategies for a substance ontologist to accommodate the Parthood View,^18^ Kingma (2018: 176) emphasises that the ‘Beginning-at-birth’ view has some attractions: it is “numerically neat: it is clear how we count organisms” and “it marks out birth as a substantial change”. However, it also comes with a huge drawback: it is simply not plausible, Kingma complains, that there should be no numerical identity between the neonate or baby^19^ and the foster: “Surely the new mother is holding in her arms the very *thing* she was pregnant with?” (Kingma, 2018: 178; emphasis in the original). In the light of this, Kingma (2018: 179ff.) considers a possible revision of the Parthood View. Could one not reject the orthodox view that substances cannot have substances as parts? Thus, in contrast to what could be called the ‘Parts-of-Substances’ Parthood View, the revised version of the Parthood View—let’s call it the ‘Substances-as-Parts’ Parthood View—holds that the foster is both a part of the gravida and a substance in its own right. The ‘Substances-as-Parts’ Parthood View secures numerical identity between the neonate and the foster insofar as substances are capable of persisting through time. Hence, we may reasonably assume that the foster, qua substance in its own right, persists through birth.^20^

The ‘Substances-as-Parts’ Parthood View faces, however, a number of difficulties. To begin with, on this view it becomes unclear how we can distinguish between body parts that are substances and body parts that are not substances. What, if any, is it that makes the foster in my womb a substance and, hence, an organism as opposed to, say, my heart and my left big toe? Note that we cannot appeal to standard characteristics of a substance, such as ontological independence, separateness and boundedness, since, as we have seen, these are exactly not possessed by the foster according to the Parthood View. The foster, we had been told, fails to be a biological individual in its own right on all relevant criteria, because of its deep and multifaceted entanglement with the gravida. Thus, all we are left with as a mark of substancehood on the ‘Substances-as-Parts’ Parthood View is the foster’s capability to persist through change and, as Kingma (2019: 619f.) emphasises, through separation. However, this capability is—according to Kingma—possessed not only by fosters, but also by organs and other parts of the body: “[…] kidneys, spermatozoa, milk teeth and hair can all become substances in their own right” (Kingma, 2018: 182f., see also 2019: 620). As a consequence, organs and other “bits of our body” (Kingma, 2018: 182) appear to be substances and, hence, organisms too.

Having to accept that on the ‘Substances-as-Parts’ Parthood View the distinctions between fosters, organs and organisms collapse is a high price to pay and demands a justification that is substantial enough to dispel the impression of an ad-hoc manoeuvre. One may also more generally wonder how convincing a version of substance ontology is, according to which ultimately just any old chunk of matter, and any old collection of chunks of matter, qualify as a substance. To be clear: a traditional substance ontologist who with Aristotle assumes that substances are composed of functional rather than mereological parts^21^ rejects all three assumptions essential to the ‘Substances-as-Parts’ Parthood View: (1) that substances can have functioning detached parts, such as kidneys (see Kingma, 2018: 181f, 2019: 620); (2) that such alleged functioning detached parts are substances in their own right just in virtue of the detachment, i.e., of the fact that they exist on their own (see also Smith & Brogaard, 2003: 47); and (3) that they have been substances already when they were still parts of an organism.^22^ Compared to the rich notion of a substance in traditional (neo-) Aristotelian substance ontology which entails complex ideas on the relationship between form and matter, whole and parts, actuality and potentiality, etc.,^23^ the notion of a substance invoked by the ‘Substances-as-Parts’ Parthood View looks rather thin, deflationary and uninformative. It is not clear what we learn about fosters when being told that they are ‘substances’. This may well be taken to curtail more generally the attractiveness of the weak, excessively permissive type of substance ontology upon which the ‘Substances-as-Parts’ Parthood View draws.^24^

There is a further difficulty. When, according to ‘Substances-as-Parts’ Parthood View, do mammalian organisms start to exist? Kingma argues that the answer depends on where one draws the spatial boundaries of the foster. If the foster is thought to comprise only those parts that will emerge as the ‘future baby’, its existence can be assumed to begin at 16 days after conception; if the foster, however, comprises umbilical cord, placenta and the entire content of the chorion (including the ‘future baby’), then its existence can be assumed to start 4 to 5 days after conception (Kingma, 2018: 183ff.).^25^ Accordingly, it would seem that either from 16 days after conception or from already 4 to 5 days after conception onwards, given that the foster is supposed to be a substance in its own right, there are two individuals where we see a pregnant individual.^26^ However, the fact that the foster is supposed to be a part of the substance which is the gravida exactly rules this out because parts of substances do not count extra. Hence, there would be only one individual where we see a pregnant individual. Kingma (2018: 181) is therefore right to admit that there is no consistent way of counting pregnant individuals on the ‘Substances-as-Parts’ Parthood View.^27^

The ‘counting problem’ (Kingma, 2018: 181) testifies to the fundamental dilemma faced by the ‘Substances-as-Parts’ Parthood View: either the claim that the foster is a substance in its own right doesn’t tell us anything interesting about the foster due to a deflationary notion of substance so that it in fact comes down to a mere stipulation of numerical identity between the foster and the neonate (as opposed to an explanation of why there is such numerical identity); or this claim, by entailing a more robust commitment to substancehood, with implications of ontological independence, separateness and boundedness, directly contradicts the parthood claim which exactly denies the foster’s possession of these characteristics.^28^ If as Kingma argues, the four criteria of the delineation of organisms—(1) homeostasis and physiological autonomy; (2) metabolic and functional integration; (3) topological continuity; (4) immunological tolerance—are not met by the foster but only by the foster-gravida whole, this making the foster a part of the gravida, how can the foster at the same time be an organism itself? As long as one stays committed to the idea that organisms are substances, one simply can’t have it both ways.

This dilemma is different from the one Kingma (2018: 179) identifies for the Parthood View, namely that the Parthood View can avoid “the counterintuitive implication that mammalian organisms cannot start before birth” only at the “cost” of accepting “a different counterintuitive implication instead, which is that organisms can be part of other organisms of the same kind”. My point is that what Kingma presents here as the second horn of this supposed dilemma (the ‘Substances-as-Parts’ Parthood View, in my terminology) is not even a coherent option. Consequently, if the first horn (the ‘Parts-of-Substances’ Parthood View, in my terminology) is unacceptable because of its exclusion of numerical identity between foster and neonate, it follows that the Parthood View as such is unacceptable—at least if it operates on the assumption that organisms are substances.

## An alternative: the process view of pregnancy

### Process ontology: organisms as processes

The Parthood View of pregnancy aims to supersede the Containment View on the basis of an appreciation of scientific findings on fosters, gravidae and their relationship. However, as we have seen, exactly those empirical facts that speak against the Containment View and in favour of the Parthood View become ultimately problematic for the latter itself: as soon as one tries to combine (1) the claim that the foster is *a part of the substance* with which it has been shown to be entangled physiologically, functionally, topologically and immunologically, i.e., the gravida, and (2) the assumption that the foster is *a substance itself* so as to satisfy the natural assumption of numerical identity between the foster and the neonate, crucial distinctions—between fosters, organs and organisms—collapse and counting pregnant individuals becomes impossible.

When discussing the Containment View, we started wondering already whether the breakdown of the Containment View in the light of science could have negative implications for its founding ontological framework. This question now resurfaces, and turns out to be even more pressing, with respect to the Parthood View: How good a match is substance ontology—an ontology that assumes ontologically independent, neatly bounded discrete things as building blocks of reality—for a theory of pregnancy that emphasises the ontological entanglement between the gravida and the foster? Is the Parthood View’s claim to superiority over the Containment View not compromised from the outset by the fact that the Parthood View works with the same basic ontological assumptions as its competitor? Could there be an alternative ontological framework—one that is better suited for a convincing account of the specific way in which the foster is ‘a part of’ the gestating organism?

In what follows, I argue that the ontology that can serve the Parthood View’s needs is process ontology. The Process View of mammalian pregnancy that I want to propose avoids the difficulties encountered by the Parthood View by doing away with the assumption that organisms are substances, assuming instead that organisms—pregnant and non-pregnant—are processes. It hereby can draw on latest empirical and theoretical work on the nature of organisms.

In the light of recent advances especially in systems biology, developmental biology and their fusions with evolutionary biology (‘evo-devo’) and ecology (‘eco-devo’) (Alberghina & Westerhoff, 2005; Barresi & Gilbert, 2019; Boogerd et al., 2007; Gilbert & Epel, 2015; Green, 2017; Noble, 2006; Nuño de la Rosa & Müller, 2021; Robert, 2004), a growing number of philosophers of biology, biology-inspired metaphysicians and biologists (Bapteste & Dupré, 2013; Bickhard, 2011; Dupré, 2012, 2020; Jaeger & Monk, 2015; Meincke, 2018b, 2019a, c, 2020b, forthcoming a, forthcoming b; Nicholson & Dupré, 2018; Simons, 2018) are promoting process ontology as the most appropriate ontological framework for research on life and living beings in science and philosophy. This process ontological turn is driven by the endeavour to do justice to the all-pervasive dynamicity of biological reality.

Undeniably, evolution is a macroscale process that creates ever new life forms and species. As we now know, developmental processes co-shape this process together with genetic factors as organisms strive to adapt to their environments. Note that development is persistent: biological lives unfold dynamically in characteristic patterns—in ‘life cycles’ with different, often radically different, developmental phases.^29^ Moreover, at any given time, an organism, qua dynamical system, exists only thanks to its own efforts: its activity is its being. Crucially, activity necessarily takes the form of interaction—interaction among the various elements within the organism’s internal environment including its numerous microbial symbionts^30^ as well as interaction with the external environment. The arguably most fundamental kind of interaction is metabolism. Only if a constant exchange of matter and energy between internal and external environment takes place, stability far from thermodynamic equilibrium can be maintained. Organisms persist by metabolising—they die as soon as metabolism breaks down. Process and change thus are at the heart of the existence and identity of organisms.

While the main idea behind the ‘process biology’ movement is clear enough, how the details are spelt out varies considerably.^31^ The same diversity of perspectives characterises the history of process thinking as a whole.^32^ This in part reflects the fact that, concerning the basic metaphysics, there is anything but agreement—not even among self-declared unconditional advocates of process ontology—on the nature of processes and their relationship with (hypothetical) things or substances (Meincke, forthcoming a). I should therefore at least indicate^33^ what kind of process ontological framework the Process View of Pregnancy invokes before outlining how the Process View facilitates a novel and more constructive way of thinking about pregnant individuals.

The process view of life presumed here starts from the metaphysical concept of a ‘process’ as an entity for whose identity change is essential—as opposed to a ‘thing’ which is an entity for whose identity change is not essential. The wording of these definitions must be taken seriously. To claim, as process philosophers do, that processes in the defined sense are ontologically fundamental is not tantamount to claiming that the world is a chaotic place without structure. The idea rather is that whatever structure—‘identity’—exists arises through change and thus is processual in essence. This idea traces right back to Heraclitus, the first process philosopher on record in Western philosophy. Heraclitus was famously reported by Plato to have taught “that all things pass and nothing stays, and comparing existing things to the flow of a river, [Heraclitus] says you could not step twice into the same river” (Plato, 1997, *Crat.* 402a; see Graham, 2019). But what Heraclitus in fact said seems to be rather this: “On those stepping into rivers staying the same other and other waters flow” (Graham, 2019). Surely, a river that does not flow, i.e., does not change the waters it consists of, is no river. So processes change and, through changing, remain the same over time—they *stabilise* in certain respects and at certain time scales (possibly to such an extent that we may mistake them for things).

Turning now to those particular processes that are investigated by biologists—‘living processes’—I am going to assume for the sake of argument the following five claims, which I have defended and laid out in more detail elsewhere (Meincke, 2018b, 2019a, c, 2020b, forthcoming b): (1) Living processes display varying degrees of unity and stability as they emerge within a dynamic and complex network of processes through processes of interactive self-stabilisation of various kinds. (2) Organisms are complex higher-level living processes that are constituted by, and not identical with, lower-level living processes. (3) This constitution relation can be more specifically described as one of *autopoiesis* (‘self-production’): an organism ‘produces itself’ by producing the lower-level component processes that in turn produce the higher-level process which is the organism (see Maturana & Varela, 1980: 78f.),^34^ with production in each case being facilitated through interaction with the environment^35^ and entailing an irreducibility of the higher-level process to its lower-level processes or ‘parts’^36^. (4) An organism’s identity over time, hard-won through autopoietic interactive self-stabilisation, is intrinsically dynamic and processual.^37^ (5) Speaking of an organism’s synchronic identity involves an abstraction from that organism’s diachronic identity since there is no change, no process, at an instant.

### Pregnant organisms: asymmetrically bifurcating hypercomplex processes

Assuming a process view of life along the lines indicated, what should we say about pregnant individuals then? Here is my suggestion: the gestating organism is best understood as a process that is in the process of splitting or bifurcating: an *asymmetrically bifurcating higher-level process*. More precisely, a gestating organism is a ‘hypercomplex’ higher-level autopoietic process that incorporates and actively maintains, through processes of both mutual stabilisation and successive disentanglement, an internal, dynamical and asymmetrical relation with an organised complex of lower-level processes that is potentially or rudimentarily a higher-level autopoietic process of the same kind of realisation. Let me unpack this dense statement.

A gestating organism is a *hypercomplex* higher-level autopoietic process in that its higher-level complexity is not sufficiently explained in terms of an autopoietic interdependence relation with lower-level processes because it involves a specific cross-level diachronic complexity, resulting from bifurcation.^38^ This diachronic complexity—the organism’s developmental profile—is synchronically relevant; and this not only in the general sense mentioned earlier that, qua process, an organism cannot ontologically be reduced to an instant in time. There is additionally a rather specific sense in which the diachronic dimension matters: one doesn’t understand what a pregnant organism is while abstracting away from the fact that this organism—a higher-level autopoietic process—produces^39^ more than just itself; that it, in this sense, consists of more than just itself. A process of producing another higher-level autopoietic process of the same kind of realisation is embedded in the autopoietic process of inter-active self-production that is the gestating organism.^40^

To say that a higher-level autopoietic process is being produced is to say that this process is *not yet higher-level* autopoietic. That is, this process does not yet possess the capability to ‘produce itself’ through interactions with its environment, thereby drawing on lower-level component autopoietic processes that subordinate their autopoiesis to the autopoiesis of the higher-level autopoietic process (Maturana & Varela, 1980: 107ff.); it possesses this capability only potentially or rudimentarily. Still, it is important to emphasise this *potential or rudimentary higher-level* character of autopoiesis because this distinguishes the ‘fostal’ organised complex of processes ontologically from its—and the gravida’s—lower-level autopoietic components, such as cells and organs. We also add that the higher-level autopoietic structure, once realised, would be of *the same kind of realisation*^41^ to highlight the difference between the foster and the gravida’s symbionts.^42^

When speaking of an *internal* relation that the hypercomplex higher-level autopoietic process incorporates and actively maintains with a rudimentarily autopoietic, organised complex of lower-level processes, this is not meant to invoke the technical sense of an internal relation, i.e., a relation that simply holds in virtue of the intrinsic natures of the relata (a so-called thin relation like ‘being taller than’; MacBride, 2016). We must not at all think of a relation that presupposes its relata as independent existents. Instead, we must envisage a relation that interactively—or (to borrow a term from Barad, 2007: 128) ‘intraactively’—produces one of its relata.^43^ The expression ‘internal’ thus refers to the hypercomplex structure of the bifurcating process that incorporates this relation *in itself*, namely as something actively brought about and thereby pushing the whole’s boundaries. As is well-known, the hypercomplex unity dilates as it bifurcates. It is insufficient to account for this in purely spatial terms, with the foster’s growth. We ought to recognise the bilateral, relational effort invested to bring about this growth within a certain time frame.^44^

This already points to the fact that the relation in question is *dynamical* in that it takes the form of action and interaction, thereby developing over time in characteristic ways.^45^ Looking at the gravida-processes, we find most importantly active integration in all the respects Kingma (2019: 622ff.) mentions when arguing against the Containment View: topology, metabolism, homeostasis, physiology, immunology.^46^ At the same time, the foster-processes actively respond to this—pregnancy is no one-way road. For instance, we now know that immune responses to maternal material tend to be missing in the first and second trimester not as a result of underdevelopment and corresponding passivity of the foster’s immune system but rather because they are actively suppressed, this changing towards the third trimester where the fostal immune system is more likely to react to foreign material (McGovern, 2017). This nicely illustrates the co-presence of processes of mutual stabilisation and of successive disentanglement: the interaction between maternal, or ‘gravidal’, and fostal processes is constitutive of both integration and emancipation, with an emphasis on the former in the beginning of the pregnancy and an emphasis on the latter towards the end of the pregnancy as bifurcation progresses.

Speaking of bifurcation, the internal, dynamic relation entertained by, and incorporated in, the hypercomplex higher-level autopoietic process is *asymmetrical* by referring to a process in which a complex of lower-level processes is splitting off from a larger complex of processes, with what is splitting off being ontologically dependent on what it is splitting off from.^47^ Ontological dependence clearly holds in the sense of *existential dependence*: for there to be a foster there must be, in the first place, an organism which gestates the foster, and the foster remains dependent on that organism. Formally:

*Existential dependence* Necessarily, the foster exists only if an organism that gestates it (a gravida) exists, and it is not the case that, necessarily, the gestating organism (the gravida) exists only if a foster exists.

Note that this is not *rigid* existential dependence: The foster could be the product of in vitro fertilisation^48^ and be gestated by a surrogate mother.^49^ (A given foster *x* is dependent for its existence on *some* gestating organism, not on a *particular* gestating organism.) Note also that this is true only on an extensional interpretation of the term ‘gestating organism’ or ‘gravida’: intensionally, the gravida *qua* gravida, too, exists only if a foster exists, namely the foster the gravida is gestating. A gestating organism is a gestating organism only if it gestates something. However, whether or not it gestates something, it is an organism, namely the same organism that didn’t gestate beforehand. Compare budding in hydra or in ordinary plants. A non-budding plant is a plant just as a budding plant, and it is the same individual plant that was not budding before.^50^ The fact that some gestating organisms die as a result of gestation does not speak against the asymmetry of the internal relation characterising the hypercomplex process. It is because of the occurrence of mutual stabilisation, i.e., because of the active integration of the foster in the gestating organism, that fosters occasionally kill the gestating organism (before, apart from exceptional circumstances, dying themselves as a result of the gravida’s death).^51^

### One or two?

Given this basic picture,^52^ how then shall we count pregnant organisms? To answer this question, we need to keep in mind that, according to the Process View, a pregnant individual is a process that is caught up in bifurcation, that is, in a transition from one to two processes. Recalling Heraclitus’s praise of rivers as possessing identity by virtue of change, consider as an analogy a bifurcating river. How many rivers are there just before we see two rivers stream downhill? One or two? One might think, because at this point there is no spatial separation, there is actually only one river. However, experts in fluvial dynamics tell us that, despite the absence of spatial separation, the currents are already starting to go separate ways.^53^ My view is that a river which is in a process of splitting is neither one river nor two rivers but both, or—if we are prepared to abolish the binary logic of substance ontology altogether—that it is neither strictly one nor strictly two rivers but something in between one and two. The same is true of the pregnant individual.^54^ If, as I have argued, a pregnant individual is a bifurcating process,^55^ then the ‘How many…?’ question applied to a pregnant individual cannot have a natural number as an answer.^56^

What about the fact that the bifurcation in the case of pregnant individuals is asymmetrical? One may think that taking asymmetry into account pushes one towards the claim suggested by the ‘Parts-of-Substances’ Parthood View that a pregnant individual is exactly one individual. If fosters are existentially dependent on the organism that gestates them and not vice versa, why should we attribute any numerical value to them, whether this be one (as claimed by the Containment View and (possibly) the ‘Substances-as-Parts’ Parthood View) or less than one (as suggested by the Process View)? However, unilateral ontological dependence as such does not entail numerical oneness. For instance, the mild climate on the British Isles is for its existence dependent on the existence of the North Atlantic Current, the northern extension of the Gulf Stream. Yet, we do not count the mild climate on the British Isles and the Gulf Stream as one thing. Arguments for the numerical oneness of foster and gravida, hence, cannot rely on asymmetry alone but must rely on further considerations. They may, as we have seen, appeal to specific biological aspects of pregnancy, such as the physiological, functional, topological and immunological intertwinement between foster and gravida.

One of the most important and obvious reasons for insisting that, despite all this, fosters somehow *do* add to the oneness of the gravida is their being offspring. To put it bluntly: if a pregnant individual were numerically one just as before it became pregnant, either something would be seriously wrong, or we would have to conclude that being pregnant had nothing to do with reproduction. Reproduction is the production of a new organism and, hence, the foster cannot be *merely* a part of the gravida, let alone a part like an organ, such as a reproductive organ.^57^ This is not to say that the foster is *not* a part of the gravida—it may be a *sui generis* part as indicated earlier.^58^ The Process View can be understood as clarifying the sense in which the foster’s being part of the gestating organism is *sui generis*: the foster is a coming-to-be descendant of both the gravida, i.e., the mother, and the father; it is a part of the gravida that is developing into a full-blown individual, meant to live its own, independent life at some point in the future.

To say that the foster is offspring is to say that there is really a bifurcation taking place in and with the gravida, making her a hypercomplex process as explained. The foster, as it were, is (in the process of) ‘springing off’ the gravida. However, as this process—the process of bifurcation, of individuation or ‘springing-off’—is not yet completed, we do not yet have a second individual. The foster is a coming-to-be individual, living in a dynamical autopoietic unity with the gravida and being existentially dependent on her: if the gravida dies, the foster most likely will die too, certainly up until 23 weeks of gestation (and in many places of the world and most of history for many weeks after). It follows that the foster is not nothing but not a full-blown individual either; and this is to say that it is something in between zero and one. A pregnant individual, therefore, is neither one nor two individuals but something in between one and two.^59^

If the emergence of a new mammalian organism does not fall into the period of gestation, when does it happen then? When is the process of asymmetrical bifurcation completed? A natural event to consider is birth. However, birth is neither just like taking the bun out of the oven, as conceived by the Containment View, nor is it some mysterious leap from one to two organisms as it appears on the ‘Parts-of-Substances’ Parthood View. Birth is rather a process itself, i.e., a phase of topological and physiological splitting within an overall process of splitting that consists of many phases; and it takes time (in humans fourteen hours on average for first-time mothers and eight hours on average for others). As a result, it is hard or impossible to pinpoint the exact moment when a child is born.^60^ ‘Birth’ is loaded with vagueness just like ‘conception’^61^ or ‘death’.^62^

But there are more reasons to doubt that birth marks the occurrence of a full-blown mammalian individual.^63^ The Blue Wildebeest’s calves can stand within an average of six minutes from birth and walk within thirty minutes; they can outrun a hyena within a day. Surely, a new-born human baby can’t do that. It takes one to one and a half years until human toddlers walk their first steps. New-born babies are entirely helpless and dependent on care. Additionally, and even more importantly, in all mammals there is continuing physiological and immunological entanglement between the baby or offspring and the mother through breast-feeding. This suggests that the bifurcation that characterises pregnancy continues, with the offspring gradually becoming more independent of the mother.^64^ Pregnancy, in other words, is not over with birth. There is something like postnatal pregnancy. Breast-feeding mother and baby, cub, puppy, foal or what have you still form a hypercomplex asymmetrically bifurcating process which is something between one and two.

## Conclusions

In this paper I have first recalled the ontological roots of the hitherto predominant view of mammalian pregnancy. The Containment View of pregnancy is steeped in substance ontology, namely in the particular version of Aristotelian hylomorphism. The ontological background explains why the Containment View traditionally co-occurs with the (logically independent) Two Individuals View. However, insofar as the claim that the foster is merely contained in the gravida is a corollary of the underlying ontological framework, which regards both foster and gravida as substances, the question arises whether the empirical untenability of the Containment View indicates that something is wrong with the ontological framework too.

Second, I have assessed the Parthood View of mammalian pregnancy which has been tentatively defended by Kingma as an alternative to the prevailing Containment View. I have argued that the Parthood View either wrongly equates fosters and organs or leaves unexplained the specific *sui generis* sense in which a foster may be a part of the gravida. The Parthood View, as I have further argued, lacks the resources to fill this gap as long as it—just like the Containment View—operates on the common presumption that organisms are substances. This becomes most evident in the ‘Substances-as-Parts’ version of the Parthood View, which aims to secure numerical identity between the foster and the neonate by claiming that the foster is both a part of a substance (i.e., of the gestating organism) and a substance in its own right. Either the claim that the foster is a substance doesn’t tell us anything interesting about fosters, or it outright contradicts the parthood claim which emphasises the multifaceted biological intertwinement of the foster and the gravida, thereby preventing a coherent way of counting pregnant individuals.

In response to this, I have then proposed a novel approach: the Process View of pregnancy, according to which mammalian pregnancy is to be understood as the gradual asymmetrical bifurcation of a hypercomplex higher-order autopoietic process. The Process View suggests a third answer to the question of how many individuals are present where and when we see a pregnant individual: a pregnant individual ‘is’ neither one nor two but something in between. This vagueness flows naturally from the processual constitution of organisms in general and of mammalian pregnancy in particular. Substances—things—observe a binary logic of ‘either-or’: either they exist, or they do not^65^; and, accordingly, either there is one, or there are two. However, for biological processes it is normal to emerge gradually and fade away gradually. Assuming a process view of life, there is no need to postulate that organisms possess sharp boundaries both synchronically and diachronically (as, e.g., Smith & Brogaard, 2003: 76 do). It is fine to admit that there is no exact time when a mammalian organism comes into existence.^66^

The Process View is able to reconciliate those two intuitions which, presuming that organisms are substances, inevitably get into conflict: the intuition that the foster, being closely connected to the gravida, must be a part of the gravida, and the intuition that the foster is numerically identical with the neonate. If organisms are processes, the latter intuition does not force us to conclude that the foster must be an individual in its own right. Instead, we can happily assume that there is a relation of identity^67^ connecting the foster and the baby, while insisting that the foster is not yet a full-blown individual because it is only just developing into one. The foster is a coming-to-be individual resulting from reproduction and undergoing gestation. This, if anything, is what makes it a special ‘lady part’.

Proponents of the Parthood View may want to claim that the Process View defended here is just a version of the Parthood View. I disagree. Once it is acknowledged that organisms are processes rather than substances, it becomes evident that the claim that a foster is a part of the gravida, while being true, fails to express what is special about fosters and their relationship to gestating organisms. To this end, one must take into account the diachronic dimension of mammalian pregnancy. The Process View of pregnancy emphasises *transition* instead of *composition* as opposed to a static analysis that ignores the characteristic developmental trajectory of pregnancy^68^: fosters are on the way towards being individual mammalian organisms and so are, as I have further argued, new-born offspring (at least of altricial mammalian species). Unlike both the Containment View and the Parthood View, the Process View captures these characteristic dynamics. It does justice to the gradual coming-into-existence of a new mammalian organism that happens through a stepwise emancipation from the gestating organism before, during and after birth.^69^

## Footnotes

1 An individual, in the metaphysical sense, is any entity that, under some sufficiently fundamental description, counts as one entity, distinct from other entities.

2 ‘Foster’ is a Danish term, which I, following Smith and Brogaard (2003), shall use to refer to the entity that is gestated by a gestating organism across all developmental stages at least from implantation.

3 Following Kingma (2019: 611), I shall use occasionally the medical term ‘gravida’ for a gestating organism. A gravida is the entity that is pregnant with a foster, with this definition not being meant to entail any particular view about the mereo(topo)logical relationship between gravida and foster. See Finn (forthcoming) for an in-depth analysis of possible views of the mereotopological relationship between gravida and foster.

4 The terminology (‘Parthood View’, ‘Containment View’) has been introduced by Kingma (2019).

5 Arguably, any material parts of a material entity *x* are contained in *x*. The Containment View of pregnancy, however, entails a negation of the Parthood View. Some doubts as to whether a sharp distinction between both views can indeed be maintained will be presented below in Sect. 3.2.

6 The exact reference of the term ‘organism’ is subject to controversial debate among philosophers of biology as is the relationship between ‘organism’ and ‘biological individual’ (Bouchard & Huneman, 2013; Clarke, 2010, 2013; Lidgard & Nyhart, 2017; Meincke & Dupré, 2020; Pradeu, 2016a, b). Before outlining my metaphysical views on organisms in Sect. 4.1, my usage of ‘organism’ will be as non-committal as possible, the working definition being something like: an organism is a living entity that, by being an organised and integrated whole, qualifies as a ‘biological individual’, i.e., as a biological entity that, under the given description, counts as one, distinct from other entities.

7 Restricting the scope of inquiry in this way is primarily heuristically motivated, given that the existing debate on the metaphysics of pregnancy centres around mammals and, more specifically even, placental mammals, which excludes marsupials and monotremes (see, e.g., Kingma, 2019: 610). The main ideas of the Process View outlined here are actually more widely applicable, which I take to be an asset; see note 69.

8 This idea pervades debates in philosophy and public discourse; see Baron (2018) and Finn (2018) for a critical discussion with respect to abortion and surrogacy.

9 Kingma (2020a) elaborates on her discussion of these—broadly physiological—criteria, taking into account criteria that correspond with an evolutionary approach to biological individuality, as suggested by Clarke (2010, 2013). She argues for a mixed verdict, according to which a pregnant organism, i.e., the foster-gravida whole, can be thought to meet some of the relevant evolutionary criteria of biological individuality, such as possessing policing devices and undergoing, as a whole, natural selection, but fails to meet others, such as having a homogeneous genotype, being the mitotic product of a sexually fertilised zygote and being the mitotic product of a bottleneck stage in the lifecycle. In contrast, Grose (2020: 1050) argues that an evolutionary account of pregnancy “yields the clear verdict that there are two organisms”. Nuño de la Rosa et al. (2021) approach the question of biological individuality in pregnancy from a different perspective that draws on evo-devo studies of eutherian reproduction, and conceptualise pregnant females as so-called historical individuals.

10 Mothers certainly have a story to tell about this. Having been through nine months of pregnancy and typically eight to fourteen hours of giving birth will make it even harder to believe that pregnancy amounts to mere containment and that birth is “the mere passage of an entity from one environment to another ([…] analogous to an astronaut leaving her spaceship)” (Smith & Brogaard, 2003: 65). For more on birth see Sect. 4.3 of this paper.

11 This is *not* to deny a contribution towards its own biological development on the part of the foster, maybe even in the sense of an ‘active agency’, as one of the anonymous referees has suggested to me. In fact, in previous work I have argued in favour of such a contribution, as being facilitated by certain capacities for self-organisation possessed by the foster, and manifesting itself in a continuous process of successive acquisition of ever-new dispositions towards the next developmental step (Meincke, 2015, 2018a). Importantly, however, this process, qua being interactive, is highly context-sensitive—as opposed to both Aristotle’s idea of development as the unfolding of an essential form’s inherent plan or ‘entelechy’ and its neo-Aristotelian remakes that identify Aristotle’s entelechy with a genetic programme supposedly determining from the outset the foster’s developmental path in its entirety. See Sect. 4.2 of this paper.

12 These do not even exhaust all familiar senses in which something can be a part of something. Consider statements like ‘The actors are parts of the play’ or ‘A fireplace is part of every decent English house’.

13 An astonishing exception are the female swamp wallabies (*wallabia bicolor*). Being blessed with the possession of two uteruses, they conceive new offspring during an ongoing pregnancy and end up being pregnant continuously.

14 This holds notwithstanding the fact that female reproductive organs (just like male ones) undergo substantive changes in the course of sexual maturation.

15 In case there are, as one anonymous reviewer has suggested, shorter-lived organs related to development and reproduction, possibly with immunological function, my argument concerning the difference in temporal profiles will be restricted to the ‘textbook’ reproductive organs mentioned above. Of course, it would need to be shown that those hypothetical shorter-lived (immunological) organs indeed would qualify as organs. If they did, note that their temporal profile would still be likely to differ from the temporal profile of pregnancies typical of the respective species (e.g., 9 months for humans, 11–12 months for horses, up to 22 months for elephants, etc.).

16 Recent research appears to contradict this traditional opinion, pointing towards the appendix functioning as a haven for beneficial bacteria.

17 The basic idea shared by the various arguments for this latter claim is that for the foster to qualify as a substance, its unity must be strong enough to rule out fission or fusion, in particular twinning or chimerism. This idea has proven influential for debates in metaphysics on personal identity, especially in the context of so-called animalist theories, which assume biological identity criteria for human persons. Animalists standardly claim that ‘we’ do not start to exist before twinning ceases to be possible (Olson, 1997: 90ff.; van Inwagen, 1990: 152ff.; Wiggins, 2001: 239). For a critical discussion of this ‘Fourteen Days Claim’, see Meincke (2020b).

18 Kingma does not endorse a substance ontological framework, at least not the one proposed by Smith and Brogaard (2003) (see Kingma, 2018: 167). All she claims to be committed to is a “common-sense ontology” which “countenance[s] organisms, kidneys, and part-whole relations, and which grant[s] that kidneys are parts of organisms” (Kingma, 2019: 612).

19 In her response to Smith and Brogaard (2003), Kingma’s focus is on human embryogenesis (which is reflected in her choice of terminology, e.g., talk of ‘babies’); but she takes her considerations to be applicable to mammals more generally (see Kingma, 2018: 166; for some caveats, see 166n4).

20 Given the temporal restrictions to the persistence at least of *material* substances, and given that no Aristotelian appeal to a natural kind ‘form’ and an ‘intrinsic potential’ appears to be doing the work in the background, additional arguments would actually be needed to support of this claim. In principle, the thesis that a foster is a substance (whether being a part of another substance or being merely contained in another substance) could be combined with the thesis that this substance is replaced with a numerically different substance at birth, e.g., in the human case with a baby or child.

21 As Scaltsas (1994: 83ff.) rightly points out, functional parts are identity-dependent on the whole they are parts of; they are unified by a substantial form. In contrast, mereological parts are identity-independent of the mereological sum they are parts of, which is to say that a mereological sum is not a substance. A substance, qua unified whole, ‘is more than the sum of its parts’.

22 To say that a substance is composed of functional rather than mereological parts is to say that (at least as far as an organism’s heteromerous parts, i.e., the organs, are concerned) there are no functioning detached parts of substances—a detached finger is called a finger only homonymously, as Aristotle puts it (2004, *Met*.1035b 25). This claim finds empirical support in the fact that organs, such as a kidney, can continue to function outside an organism only with sophisticated technological means and only for a limited time—eventually they need to be implanted in an organism. Evidently, if there are no functioning detached parts of substances, there is no need to speculate about the diachronic identity of such parts. Note that it remains open what, if anything, follows from this for the status of the foster, given that, as I have argued in the previous section, fosters cannot unproblematically be compared to organs. Kingma (2019: 619f.) argues that a type of substance ontology which denies that substances can have substances as parts is likely to deny the foster’s persistence through birth to the extent that it denies an organ’s (e.g., a kidney’s) persistence through transplantation. This strikes me as questionable. At least traditional (neo-) Aristotelian ontologies are absolutely fine with granting persistence through birth to the foster while denying persistence through transplantation for organs because these ontologies do exactly not subscribe to an analogy between fosters and organs.

23 Kingma nowhere acknowledges the traditional Aristotelian background of the Containment View of pregnancy. This leads to somewhat futile speculations about why proponents of the Containment View, such as Howsepian (2008), standardly deny that humans can be parts of humans (Kingma, 2019: 616ff.). Only briefly the possibility is considered that this denial is to be explained through “a more general metaphysical view on which substances can’t have parts that are themselves substances, which would then be capable of persisting through their separation” (Kingma, 2019: 619f.), but this view is not identified as Aristotelian, i.e., as having its roots in hylomorphism. Instead Kingma conjectures: “Presumably this view invokes some special or restricted understanding of ‘substance’. For cells, molecules and atoms are all good candidates for being substances, on at least one natural understanding of ‘substance’” (Kingma, 2019: 620).

24 Kingma (2020b: 383, 2018: 175 and 179f.) refers to the version of substance ontology defended by Hoffman and Rosenkrantz (1994) as well as to elements in Smith and Brogaard (2003) in this regard.

25 Here Kingma presupposes a revised version of Smith and Brogaard’s (2003) substance ontological framework and draws on considerations on causal unity, boundaries and the possibility of twinning with a focus on human pregnancy (see note 19).

26 From day 16 onwards, there might be even three individuals present where we see a pregnant individual if we take seriously the possibility considered by Kingma (2018: 185) that the ‘future baby organism’ and the ‘chorionic content organism’ co-exist, with the former being a part of the latter and, unlike the latter, surviving birth.

27 Note that we cannot resort to the claim that parts of substances do not *normally* count extra but that in this particular case—the case of the foster—a substance’s part *does* count extra because this part is itself a substance. If, as discussed above, the status of a substance cannot be reserved to the foster but, according to the available criteria, is possessed also by the other parts of an organism, then either these other parts will count extra too or no part of the body (the foster included) will count extra. In other words: the use of a deflationary concept of substance gives away any possible resources for maintaining a *sui generis* character of the sense in which the foster is a part of the gravida, including a hypothetical *sui generis* way of counting.

28 The ‘Substances-as-Parts’ Parthood View then threatens to collapse into the Containment View which, too, holds that fosters are substances, but denies that fosters are parts of gravidae exactly because of their status as substances and, i.e., because of the fosters’ supposed ontological independence and topological separateness, which standardly is taken to be grounded in the foster’s instantiation of a particular form or natural kind.

29 This is not to deny the possibility of distinguishing (not for all but at last for some species) immature from mature stages of development (see Nuño de la Rosa, 2010). But it is to emphasise that any existing mature stages are not static ‘endpoints’ of development but continue to develop in characteristic ways.

30 Organisms are so-called holobionts: “anatomically, physiologically, developmentally, genetically, and immunologically multigenomic and multispecies complexes” (Gilbert et al., 2012: 331).

31 See Nicholson and Dupré (2018) for a comprehensive though not exhaustive collection of views.

32 See Seibt (2020) and Rescher (1996) for helpful overviews.

33 For fuller presentations of the fundamental metaphysical questions at issue and my take on these, see Meincke (2018b, 2019b, c, 2020a, forthcoming a, forthcoming b).

34 An autopoietic system is “organized (defined as a unity) as a network of processes of production (transformation and destruction) of components that produces the components which: (i) through their interactions and transformations continuously regenerate and realize the network of processes (relations) that produced them; and (ii) constitute it […] as a concrete unity in the space in which they (the components) exist by specifying the topological domain of its realization as such a network”. For a detailed demonstration that the autopoiesis theory implies a process view of life, see Meincke (2019a); also Meincke (forthcoming b).

35 That an autopoietic system is not a *causa sui* but remains dependent on interaction is important to acknowledge to avoid substantialist misinterpretation, see Meincke (2019a: 8ff.).

36 Any part’s function and existence flow from the processual organisation of the organism as a whole as much as the parts themselves generate the whole, see Meincke (2019a: 4ff.).

37 For latest perspectives on biological identity from both metaphysics and the philosophy of biology, including process ontological approaches, see Meincke and Dupré (2020).

38 Bifurcation, in other words, is not local but its dynamics cut through the hierarchy of organisational levels.

39 ‘Production’ is an appropriate choice of terminology. Organisms both ‘self-produce’ and ‘reproduce’, the latter by producing a new (ultimately self-producing) organism of the same kind. In the case of sexual reproduction, as found in mammals, this implies, of course, that fathers are co-producers of fosters. However, their contribution is confined to genetics and to conception, i.e., the initiation of pregnancy. The pregnancy-part of production of the new organism is carried out by mothers. It is of crucial importance, ontologically and otherwise, to recognise the productive nature of this part of ‘reproductive labour’ of females, instead of reducing their contribution to being mere ‘incubators’. The latter would take us straight back to the Containment View.

40 Pregnancy is autopoietic autopoiesis-poiesis and thus a form of top-down allopoiesis, as opposed to the bottom-up allopoiesis of lower-level autopoietic processes.

41 According to Maturana and Varela (1980: 76), the autopoietic organisation “is common to all living systems” but differently realised. Note that by appealing to potential or rudimentary higher-level autopoiesis we can leave open the question of whether the foster *already is* a member of the species of which the gravida is a member, thus bypassing a possible problem arising from the common assumption that species membership implies organism status and, hence, individuality; see Kingma (2019: 616ff.). The crucial point is that a member of the species to which the gravida belongs is *in production*.

42 This is not to deny the vital role microbial symbionts play in facilitating pregnancy, see Gilbert (2014) and Takeshita (2017).

43 That is to say, the situation is, expressed in Maturana and Varela’s terminology, not one of ordinary ‘coupling’ between two lower-level autopoietic systems, which retain their identities at the level of their interaction while giving rise to a new unity at a higher level (Maturana & Varela, 1980: 107). Though we can distinguish between fostal and gravidal interaction partners at a lower level, and though there is a sense in which a higher-level unity can be said to arise from this interaction—the gravida as hypercomplex process –, it ought not to be ignored that the interaction is targeted at fostering one of the partners—the foster –, thereby relying on, and being enacted by, the gravida as a whole (as opposed to local complexes of lower-level processes). The gravida, qua hypercomplex process, engages in a process of self-differentiation as it ‘intra-acts’ with a part of itself which it thereby (co-) produces. See also the following discussion of asymmetry.

44 Extending one’s outer limits when pregnant, in other words, is more than simply growing due to a body part’s growth; and the internal gravida-foster relation explains why. It marks the difference between complexity and hypercomplexity.

45 See Martinez Quintero and De Jaegher (2020) for a biological and phenomenological analysis of foetal and maternal agency and their dynamical interrelations.

46 See Howes (2007, 2008) for a scientifically informed discussion of the immunological relationship between gravida and foster that reveals the generally cooperative character of their interaction and emphasises the active and constructive contributions the gravida makes towards pregnancy, in contrast to the predominant immunological ‘foreign-foetus model’, according to which the gravida is antagonistically inclined towards the foster and prevented from expelling it only through a separating immunological barrier. Nuño de la Rosa et al. (2021) offer a compelling critique of common accompanying hypotheses on the evolution of pregnancy such as ‘invasive placentation’ and ‘parent–offspring conflict’ due to conflicting genetic interests.

47 If the ontologically dependent part of the bifurcating hypercomplex process bifurcates itself (so-called twinning), we get two fostal processes jointly participating in the overall process of asymmetrical bifurcation. See Meincke (2020b).

48 In virtue of their (non-rigid) existential dependence on a gravida, fosters (in utero embryos and in utero foetuses) are (at least post implantation) ontologically different from IVF embryos, even though the latter ontologically depend for their *future* existence on some future gravida that will gestate them—at least this is the current state of the art in reproductive medicine.

49 The foster’s ontological dependence on the gravida thus may, but need not, entail causal dependence (the latter, in accordance with the received view, understood as a diachronic relation). Note that I thereby (and in contrast to the received view) do not draw a sharp line between causal dependence and ontological dependence. Instead, I am assuming that all cases of causal dependence count as ontological dependence but not vice versa.

50 Arguably, the foster is ontologically dependent on the gravida also in the sense of *identity* or *essential dependence*—to be a foster means to be gestated by another organism, i.e., by a gravida, which on some readings entails existential dependence (Tahko & Lowe, 2016).

51 In emphasising asymmetry, my processual analysis of the relation between gravida and foster differs from Martinez Quintero and De Jaegher’s (2020: 7) portrayal of pregnancy as an ‘emergent relational organisation’ which entails that *both—*“maternal body and embryo”, qua “processes of individuation”,—“depend on this relation for their existence as long as the relation holds”. Note that asymmetry does not hinder conceiving of the foster-gravida relation as constituting a new metastable state (Martinez Quintero & De Jaegher, 2020: 2, 7n9) or “a novel homeostatic state defined at the relational level” (Nuño de la Rosa et al., 2021: 7). Interactions in which one of the interaction partners takes the lead can be perfectly stable—‘stable’ in the processual sense of being the result of continuous stabilisation through interaction. In the present case of pregnancy, this stability, due to its bifurcating dynamics, is a temporary one.

52 For reasons of space and focus, I confine myself to articulating the key ideas of the Process View of pregnancy. The present paper’s central aim is to motivate this view; explicating the details is a separate task for future work.

53 Streamlines diffluence, emerging from a complex process of interaction between various factors (such as flow velocity, slope, sediment load, material properties of the riverbank, other geomorphological features, and so on) appears actually to be a cause of river bifurcation, with the exact mechanisms being subject to ongoing research. Moreover, bifurcation is a natural thing to do for rivers (something the pervasive regulation of rivers makes us easily forget), and it tends to be asymmetrical. Rivers are, as it were, organised for (asymmetrical) bifurcation—which is, of course, *not* to say that the internal relation between the dividing flow dynamics in a bifurcating river is *just the same* as the internal relation between fostal and gravidal processes in the gravida qua hypercomplex process. Identity is not to be asked of an analogy. I am inclined to think that the details about fluvial dynamics just mentioned nicely support, but are not essential to, the central point of the analogy that concerns vagueness in counting due to bifurcation. Thanks to an anonymous referee for prompting me to clarify this.

54 Without thereby claiming that rivers bifurcate *in exactly the same sense* in which a pregnant individual bifurcates (see note 53), I want to stress that bifurcation is both spatial and temporal in rivers and pregnant individuals alike. This holds notwithstanding the fact that bifurcating rivers typically keep both their pre-bifurcation and their post-bifurcation parts in existence at least for some time, whereas the pregnancy-type of bifurcation, once completed, can no longer be said to possess any pre-bifurcation parts. Pregnancy processes abandon their earlier stages as they progress, which I take to imply that pregnant organisms, just as organisms in general, are continuants, on the assumption that continuants must not be equated with substances but can be processes (for a detailed defence of the possibility of processual continuants, see Meincke (forthcoming a); see also already Meincke (2018b: 374n45, 2020b: 272n25).) I am grateful to Alexander Geddes for pressing me on this.

55 Similarly, Dupré (2020: 161) suggests conceptualising pregnancy as “a gradual bifurcation in a flow of living process”, comparing it to sympatric speciation. However, he does not present a worked-out view. Sidzinska (2017) offers a critique of what she, on the basis of Bapteste and Dupré (2013) and a talk Dupré gave at the University of Southampton in 2015, hypothesises to be a process approach towards pregnancy. Her main objection is that this approach, by focusing on biological processes only, fails to account for the emergence of subjectivity in pregnancy. For arguments as to why and how exactly a bio-processual approach (‘processual animalism’) can explain subjectivity, see Meincke (forthcoming b).

56 This fits with the phenomenology of pregnancy which some women have reported to entail the dichotomy ‘one or two’ being blurred (Tyler, 2000; see also Sidzinska, 2017). Howes (2008: 262ff.) provides interesting empirical evidence from immunology in support of a ‘not-one-but-not-two model’, according to which “[m]other and fetus are neither two distinct individuals, nor do they count as one distinct individual” (2008: 248). In contrast to these uses and to my own, Martinez Quintero and De Jaegher (2020: 3) use the ‘not one, not two’ phrase to refer to their different interpretation of pregnancy as “a new individuating process, in which its participants relate and all elements of which constitute each other” (see note 51), following Varela’s (1976) dialectic method of dissolving dualities by understanding how the elements of the duality (Varela was concerned with ‘mind’ and ‘body’), at the same time, are two (because they exclude each other) and one (because they depend on each other).

57 To have reproductive organs is a necessary but not a sufficient condition of reproduction, see Sect. 3.1 of this paper.

58 See, again, Sect. 3.1 of this paper.

59 In case of twin pregnancies, the pregnant individual is accordingly something between one and three; without this, however, implying that it is exactly two individuals. A principle of non-additivity applies (see Meincke, 2020b). Thanks to Katherine Hawley for pressing me on this point.

60 Looking at humans, is it when the baby is fully outside the mother’s womb? But at this point the baby’s metabolism is still connected to the mother’s via the umbilical cord. Is it when the umbilical cord is cut off? But in line with the latest WHO advice, the clamping of the umbilical cord today is often delayed up until three minutes after vaginal delivery. At this time the baby will already be breathing, lying in its mother’s arms and possibly enjoying its first breastfeed. What about the first breath? However, the first breath is only the very beginning of a process of adaptation of the respiratory system to extrauterine life, alongside with processes of adaptation undergone by the cardiovascular system (see also Kingma, 2018: 176f.n27, 2019: 630f.), the urine system, the immune system, thermoregulation, metabolism, and so forth.

61 Conception is a complex process consisting of several phases (capacitation of the sperm, breaking through the egg’s zona pellucida, cortical reaction of the zona pellucida, second meiotic division of the egg, fusion of the sperm nucleus with the ovum, and so on).

62 Consider on-going debates about criteria of death (cardiopulmonary death versus brain death).

63 Note that by ‘individual’ I do not mean ‘monogenetic (multicellular) individual’. Mammalian individuals are holobionts (see note 30); and what Chiu and Gilbert (2015: 192) point out with respect to human childbirth applies to mammalian birth in general: birth is “the passage from one set of symbiotic relationships (primarily relationships with the mother) to another (primarily relationships with bacteria)”. See also Takeshita (2017).

64 The older the postpartum offspring, the less likely is it that the mother’s death will result in the offspring’s death as caring responsibilities can be taken over by other members of the community.

65 “The concept of substance is instantiated either fully or not at all” (Smith & Brogaard, 2003: 50). This accords with Aristotle’s doctrine that substance does not allow for intensification or debilitation (e.g., 2004, *Met*. 1030b413, 1031b114, 1032a46, 1037a1820).

66 Compare, in contrast, Smith and Brogaard’s (2003: 75; see also 48ff.) speculation that a human (or any other mammalian organism) is formed by a “unifying process [which] is almost instantaneous” rather than “by increments across a temporal interval”. Interestingly enough, the putative unifying process is said to be only *almost* instantaneous—as if there could be such a thing as an instantaneous process.

67 Unlike numerical identity as traditionally understood, this relation of identity is thoroughly processual, i.e., permits the gain and loss of properties over time instead of complying with Leibniz’s Law (Meincke, 2018b: 372ff.). As such it must also not be confused with four-dimensionalist concepts of persistence, sometimes proposed under the heading of ‘genidentity’ (Guay & Pradeu, 2016). To be clear, a four-dimensionalist analysis of pregnancy would conceive of gravida and foster as aggregates of portions of spacetime, and would conceptualise pregnancy as a case of temporary spatiotemporal overlap between these spacetime ‘worms’. Thus gravida and foster, in the four-dimensionalist picture, are two separate entities from start to finish, which flies in the face of the characteristic temporal dynamics of gestation. Process ontology as understood and implemented in this paper is sharply distinguished from both substance ontology and four-dimensionalism which turn out to be just different versions of the same type of ontology, namely an ontology that regards static existents—‘things’—as ontologically fundamental (Meincke, 2018b, 2019b, c, forthcoming a).

68 Martinez Quintero and De Jaegher (2020: 1), too, express dissatisfaction with the “static account of pregnancy” offered by both Parthood View and Containment View.

69 The basic ideas outlined here can serve to guide future work towards a comprehensive process view of pregnancy in the animal kingdom which would cover not only placental mammalian pregnancy but also marsupial mammalian pregnancy as well as non-mammalian pregnancy. Sexual reproduction in animals comes in a striking diversity of forms (oviparity, ovoviviparity, viviparity, each with numerous subtypes) with different forms and degrees of interaction (or ‘intraaction’) between gravidae and fosters. These include different forms of matrotrophy beyond placentotrophy (e.g., histotrophy or embryotrophy in sharks) which may render a gestating organism hypercomplex in the broad metaphysical sense described in this paper (while the details obviously vary across species). No less heterogeneous are postnatal interactions between gestational parents and offspring, including different forms of postnatal matrotrophy, such as dermatotrophy, which may justify classification as a form of postnatal pregnancy. Generally, from a process perspective, the distinction between pregnancy and other forms of gestation can be expected to become blurred once pregnancy is investigated in a larger physiological and evolutionary context.
